# Annual acknowledgement of manuscript reviewers

**DOI:** 10.1186/s12998-015-0074-7

**Published:** 2015-10-01

**Authors:** Bruce Walker

**Affiliations:** Editor-in-Chief, Chiropractic & Manual Therapies, ᅟ, ᅟ

## Abstract

The editors of *Chiropractic & Manual Therapies* would like to thank all our reviewers who have contributed to the journal in Volume 22 (2014).

**Ellen Aartun**

Denmark

**Lyndon Amorin-Woods**

Australia

**Annette Bishop**

United Kingdom

**Felicity Bishop**

United Kingdom

**Kate Bolam**

Sweden

**Jennifer Bolton**

United Kingdom

**Rod Bonello**

United States of America

**Marcelo Botelho**

Brazil

**Jonathan Branney**

United Kingdom

**Alan Breen**

United Kingdom

**André Bussières**

Canada

**David Byfield**

United Kingdom

**Jerrilyn Cambron**

United States of America

**Melainie Cameron**

Australia

**Carol Cancelliere**

Canada

**J. David Cassidy**

Denmark

**John Cerf**

United States of America

**Thomas Christ**

United States of America

**Chad Cook**

United States of America

**Jeffrey Cooley**

Australia

**Colin Crawford**

Australia

**Lee Daffin**

Australia

**Laura Davies**

United Kingdom

**Katie de Luca**

Australia

**Alasdair Dempsey**

Australia

**Martin Descarreaux**

Canada

**James DeVocht**

United States of America

**Aron Downie**

Australia

**Adrian Esterman**

Australia

**Roni Evans**

United States of America

**Randy Ferrance**

United States of America

**Elisa Fluess**

United Kingdom

**John Glastonbury**

Australia

**William Grant**

United States of America

**Mitchell Haas**

United States of America

**Mark Hancock**

Australia

**Jan Hartvigsen**

Denmark

**Cheryl Hawk**

United States of America

**Stephen Healey**

United States of America

**Lise Hestbaek**

Denmark

**Alison Hogg**

Australia

**Eric Hurwitz**

United States of America

**Tue Secher Jensen**

Denmark

**Asbjorn Jokstad**

Norway

**Gwendolen Jull**

Australia

**Shane Koppenhaver**

United States of America

**Dana Lawrence**

United States of America

**C.M. Janine Leach**

United Kingdom

**Charlotte Leboeuf-Yde**

Denmark

**Chris Maher**

Australia

**Jo-Anne Maire**

Australia

**Barbara Mansholt**

United States of America

**Amanda Meyer**

Australia

**Silvano Mior**

Canada

**Timothy Mirtz**

United States of America

**Tom Molyneux**

Australia

**Donald Murphy**

United States of America

**David Newell**

United Kingdom

**Mandy Nielsen**

Australia

**Nicholas Palmieri**

United States of America

**Gregory Parkin-Smith**

Australia

**Luke Parkitny**

United States of America

**Patrick Partington**

United Kingdom

**Steven Passmore**

Canada

**Stephen Perle**

United States of America

**Cynthia Peterson**

Switzerland

**Erik Poulsen**

Denmark

**Alexander Ruhe**

Germany

**Ruth Sandefur**

United States of America

**Michael Schneider**

United States of America

**William Shelton**

United States of America

**Maureen Simmonds**

United States of America

**J Keith Simpson**

Australia

**Norman Stomski**

Australia

**John Triano**

Canada

**Arianne Verhagen**

Netherlands

**Howard Vernon**

Canada

**Bruce Walker**

Australia

**Mark Webster**

United Kingdom

**Niels Wedderkopp**

Denmark

**Jan-Falco Wilbrand**

Germany

**Jessica Wong**

Canada

**Kenneth Young**

Australia

**Martin Young**

United Kingdom

